# P01-037 – Genetic analysis practice prior to FMF diagnosis

**DOI:** 10.1186/1546-0096-11-S1-A41

**Published:** 2013-11-08

**Authors:** Y Shinar, Y Berkun, A Livneh, S Padeh

**Affiliations:** 1 Heller Institute of Medical Research, Sheba medical center, Tel Hashomer, Israel; 2 Department of Pediatrics, Hadassah Hebrew University Medical Centre, Jerusalem, Israel; 3 Sackler School of Medicine, Tel Aviv University, Tel Aviv, Israel; 4 Department of Pediatrics, Sheba medical center, Tel Hashomer, Israel

## Introduction

Despite accepted clinical diagnostic criteria overruling both negative and positive genetic results, Mediterranean fever gene (*MEFV*) testing for Familial Mediterranean Fever (FMF) is regular practice.

## Objectives

This study aimed at identifying the benefits and impact of this practice.

## Methods

Previously diagnosed pediatric patients (N=681), at a tertiary pediatric FMF clinic, were stratified according to the availability of genetic results at colchicine prescription, and according to their phenotype, defined as typical or atypical at colchicine prescription. Subgroups were compared with respect to their genetic features.

## Results

At colchicine prescription, genetic results were not available for 229/681 patients (34%). A typical phenotype was significantly more common in this subgroup than in patients with genetic testing at prescription (212/229, 92%, vs. 260/452, 58%, OR=9.2 95% CI 5.4-15.6, p=0.0001). Of note, despite the high frequency of typical phenotype in this group, the rate of 2 pathogenic variants of *MEFV* was higher (61.5% vs. 49.3%, p=0.002), the rate of genetic negative FMF was lower (7% vs. 17.4%, p<0.0001), and the rate of p.M694V homozygosity tended to be higher (31% % vs. 25, a trend) in those with gene analysis available at prescription. When focusing on typical presentation alone (n=472), the distinction between the groups increased, as in the subgroup with typical phenotype plus genetic analysis prior to prescription the rates of 2 pathogenic variants **and** homozygosity to M694V were higher than in typical phenotype without genetic testing (60.7% vs. 50%, p=0.02, and 37% vs. 27%, p= 0.037 respectively).

## Conclusion

It appears that in real life most FMF patients awaited genetic testing before colchicine prescription, with particular predilection to atypical patients. This practice results in a better match between clinical and genetic diagnosis of FMF. Of interest, an overall similar distribution of *MEFV* genotypes in typical and atypical patients suggests substantial modulation of the *MEFV* genotype–phenotype correlation in atypical patients, by yet unknown factors.

## Disclosure of interest

None declared.

